# Novel Use of Transforaminal Lumbar Interbody Fusion Trial to Aid in Midline Placement of Lumbar Artificial Disc: A Technical Note With Illustrative Case

**DOI:** 10.7759/cureus.112099

**Published:** 2026-07-05

**Authors:** Syed-Abdullah Uddin, Sakeena Hussain, Sanah Ahmed, Namath Hussain

**Affiliations:** 1 Neurosurgery, Loma Linda University Medical Center, Loma Linda, USA; 2 Neurologic Surgery, Webb Schools, Claremont, USA; 3 Neurologic Surgery, Univeristy of Michigan, Ann Arbor, USA

**Keywords:** corrective spine deformity surgery, neurosurgery, neurosurgery training, spine deformity surgery, spine surgery

## Abstract

Lumbar artificial disc replacement (ADR) is an established motion-preserving surgical option for degenerative disc disease. Proper implant positioning has been associated with improved clinical outcomes, implant longevity, and reduced rates of postoperative complications. We describe a novel intraoperative technique using a transforaminal lumbar interbody fusion (TLIF) trial as a temporary lateral spacer to facilitate midline positioning of a lumbar ADR and prevent implant skiving (lateral deviation during insertion). In the case presented, repeated attempts at ADR placement resulted in lateral deviation of the implant; a TLIF trial was employed as a mechanical barrier, allowing successful positioning of the ADR. This technical note demonstrates the feasibility of utilizing a TLIF trial as an adjunctive tool to facilitate accurate coronal alignment of lumbar arthroplasty implants when standard insertion techniques prove challenging.

## Introduction

Lumbar artificial disc replacements (ADRs) have been shown to be a safe and effective means of treating low back pain and improving quality of life while maintaining lumbar motion [1-4]. Standardized workflow for insertion of a lumbar ADR includes accessing the disc space (primarily through an anterior approach), performing a discectomy, and placement of the implant with alignment/positioning confirmed through use of intraoperative fluoroscopy. Care must be taken to properly position the implant within the coronal and sagittal planes while also ensuring proper endplate coverage and adequate height. Studies have shown that by using the pedicle as a landmark, ideal placement of an ADR is within 3 mm of the interpedicular midline [5,6]. Larger deviations from the midline have been associated with higher disability scores and persistent neurologic symptoms during long-term follow-up [5,7]. Studies vary regarding ideal sagittal positioning based on device type and degree of deformity; however, there is general consensus that excessive anterior or posterior placement should be avoided [7-9]. Standard placement techniques rely on the use of direct visualization and intraoperative fluoroscopy to aid in confirmation of proper coronal and sagittal alignment. Some technical difficulty may be introduced if the implant fails to stay midline or due to the patient's deformity or angulation, prefers one side during insertion, a phenomenon commonly referred to as implant skiving. Common factors that contribute to skiving include osteoporosis contributing to a misaligned track, the vertebral endplate size and shape, and patient-specific deformity and degenerative changes [10-11]. We present a technical solution utilizing a transforaminal lumbar interbody fusion (TLIF) trial as a temporary mechanical guide to facilitate midline ADR placement in cases complicated by intraoperative implant skiving. While skiving is a well-known issue during implantation, review of the literature emphasizes accurate fluoroscopic guidance and use of image-based navigation to aid in the prevention of skiving without providing a technical maneuver to attempt to correct this common issue [4,6].

## Technical report

Illustrative case

**Figure 1:**
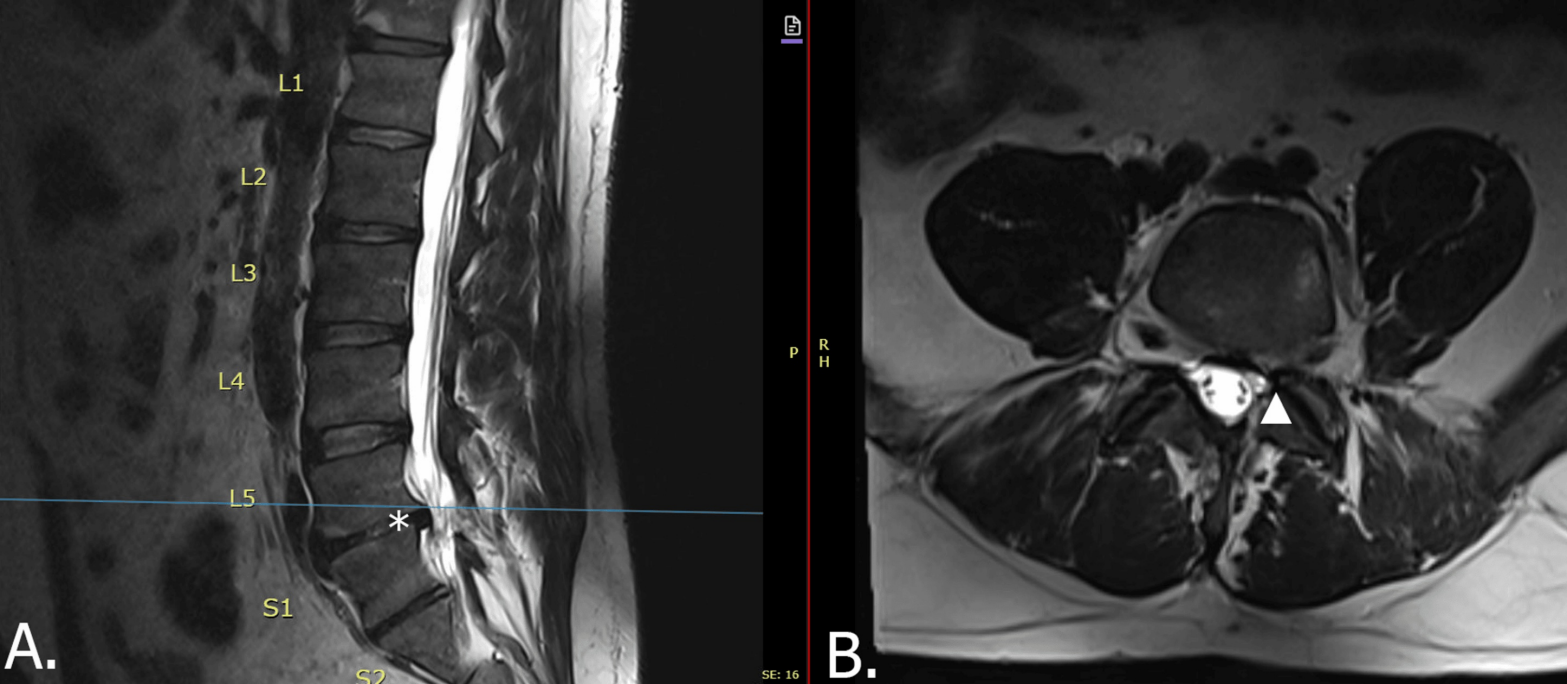
Pre-op T2-weighted MRI. (A) Sagittal image showing L5-S1 disc bulge marked with (*) causing lumbar canal stenosis. (B) Axial image showing left neuroforaminal stenosis with nerve root impingement marked with ▲.

A 36-year-old male with a history of prior L5-S1 hemilaminectomy presented with recurrent low back pain radiating into the left lower extremity. MRI of the lumbar spine demonstrated an L5-S1 disc protrusion with impingement of the descending left S1 nerve root and mild left-sided foraminal stenosis (Figure 1). The patient had previously completed several months of physical therapy with only temporary symptom relief. Neurologic examination demonstrated findings consistent with left L5-S1 radiculopathy without motor weakness or bowel or bladder dysfunction.

Following discussion of available treatment options, the patient elected to undergo L5-S1 lumbar ADR via an anterior approach.

Operative technique

The patient was positioned supine and underwent general anesthesia with total intravenous anesthesia and multimodality neuromonitoring. An anterior retroperitoneal exposure of the L5-S1 disc space was performed with assistance from the vascular surgery service due to proximity of the greater vessels.

**Figure 2:**
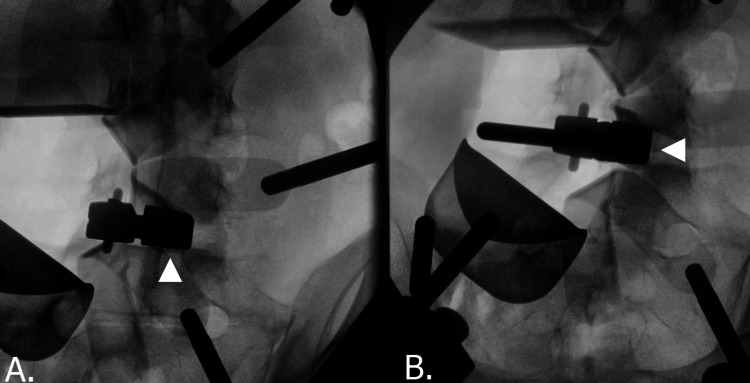
Intraoperative Fluoroscopy. (A) AP view showing proper midline ADR trial, the spinous process is used as a landmark of the midline. (B) After attempts to advance ADR lateral skive is observed away from midline (marked with ◄).

Following confirmation of the operative level using fluoroscopy, a standard discectomy and endplate preparation were completed. During insertion of the ADR, repeated attempts at positioning resulted in persistent right-sided lateral skiving of the implant despite fluoroscopic guidance (Figure 2). This malposition was directly visualized and could be observed fluoroscopically by the implant shifting away from the midline spinous process. The patient's degenerative osteophytes were thought to be contributing to the persistent skive observed.

**Figure 3:**
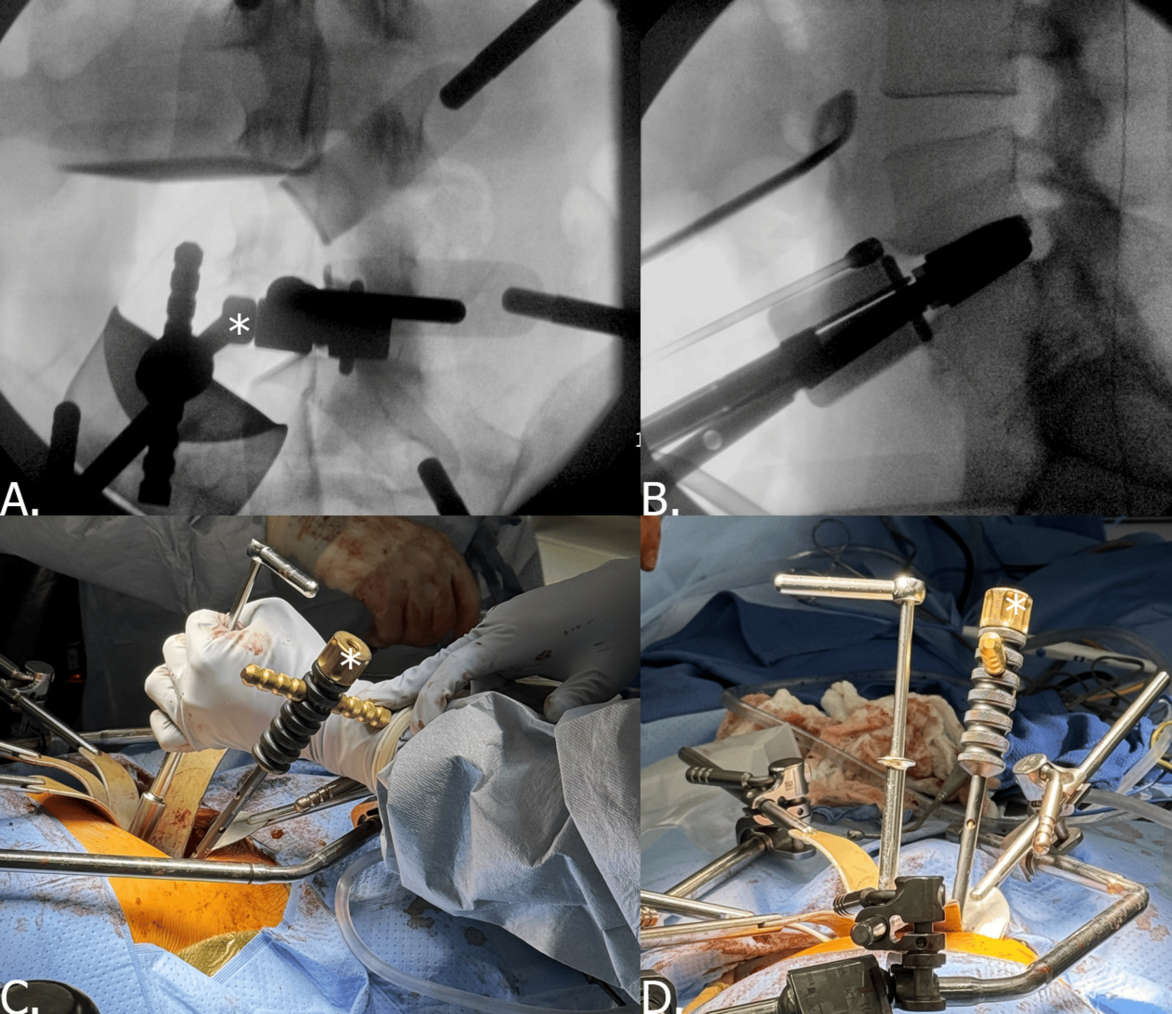
Intraoperative and Fluoroscopic images. (A) AP view of TLIF trial lateral to ADR trial to prevent skive into the lateral disc space. (B) Lateral view of TLIF trial and ADR in place showing appropriate placement in the middle of the AP axis of the vertebral body. (C) Intraoperative placement of TLIF trial (marked with *) next to the lumbar ADR placing device. (D) Standalone view of ADR-TLIF construct when aiding in placement of ADR, TLIF trial marked with (*).

To address this issue, a TLIF trial was inserted into the lateral aspect of the prepared disc space (Figure 3). The trial functioned as a temporary mechanical barrier, preventing further lateral deviation of the ADR during insertion. With the trial in place, the ADR was successfully advanced along the intended midline trajectory and positioned over the interpedicular midline, with the spinous process being used as a marker for alignment with the midline. The TLIF trial was subsequently removed, and final implant positioning was confirmed fluoroscopically.

**Figure 4:**
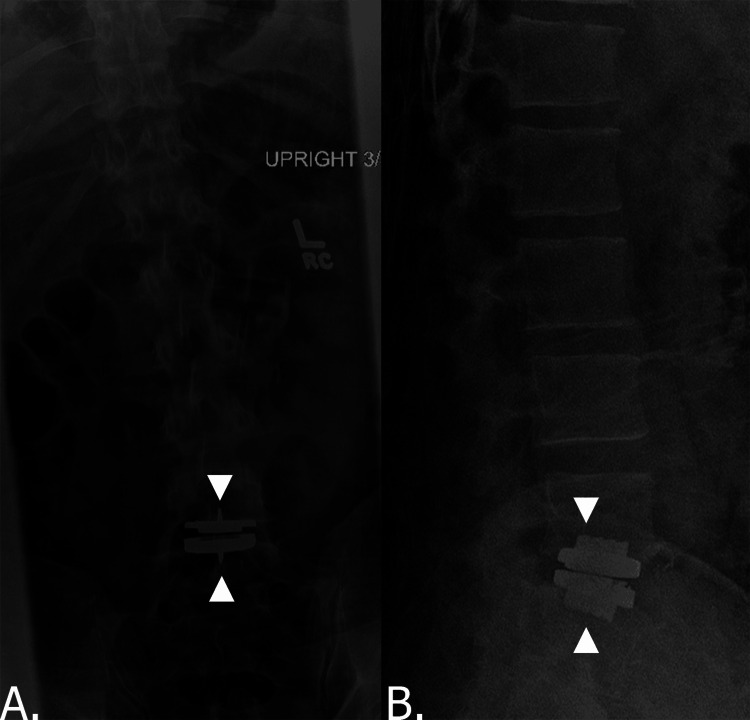
Postoperative upright X-ray images showing proper midline placement of lumbar ADR in (A) AP and (B) lateral views. The implant is marked with ▲.

Postoperative imaging demonstrated satisfactory implant alignment (Figure 4), and the patient experienced improvement in radicular and low back pain symptoms at both two-week and six-week follow-up visits.

## Discussion

The technique described herein represents a safe, straightforward, and efficient method for correcting intraoperative lateral implant skiving during lumbar arthroplasty. As demonstrated in this case with multiple failed attempts of proper ADR placement with the use of the TLIF trial, appropriate coronal alignment can be confirmed both under direct visualization and fluoroscopically. Although this technique requires a TLIF trial set to be available in the operating room, such instrumentation is standard in most spine surgery operating rooms. To our knowledge, no previously described technique has reported the off-label use of interbody trials as mechanical guides for midline ADR placement. Existing literature recommends methodical alignment and gradual implant introduction, but does not address technical rescue strategies for persistent intraoperative skiving besides surgeon judgement when replacing the implant [5,7]. While this only demonstrates a single case, given its simplicity and the wide availability of TLIF instrumentation, this approach may serve as a practical rescue strategy when standard techniques are insufficient. Pitfalls associated with this technique may include damage to the endplates, over-distraction leading to nerve root injury, over-sizing of the implant related to increased disc space size and implant misalignment, all of which were not seen in this case. In a comparative meta-analysis by de Kunder et al. that looks at TLIF and posterior lumbar interbody fusion (PLIF), it is reported that TLIF is associated with lower complication rates, reduced blood loss, and shorter operative times compared with PLIF [12].

The primary limitation of this report is that it represents a single illustrative case. No conclusions regarding efficacy, reproducibility, or superiority over standard insertion techniques can be drawn. Furthermore, the safety and long-term outcomes in patients receiving this technique have not been thoroughly explored and may be investigated in future studies. Long-term studies involving larger cohorts would be required to evaluate the frequency with which this technique is needed and its impact on implant positioning and clinical outcomes.

## Conclusions

We describe a novel technical adjunct utilizing a TLIF trial as a temporary mechanical guide to facilitate midline placement of a lumbar artificial disc replacement in the setting of lateral intraoperative implant skiving. In this illustrative case, the technique was simple to implement, required no specialized instrumentation, and enabled successful implant positioning. Although further investigation is warranted to evaluate its reproducibility, safety and clinical utility, this method may serve as a useful rescue strategy when standard insertion techniques fail to achieve satisfactory coronal alignment.
